# Specifying attentional top-down influences on subsequent unconscious semantic processing

**DOI:** 10.2478/v10053-008-0067-3

**Published:** 2009-08-21

**Authors:** Ulla Martens, Markus Kiefer

**Affiliations:** 1 Department of General Psychology I, University of Osnabrück, Germany; 2 Department of Psychiatry, University of Ulm, Germany

**Keywords:** automatic processes, unconscious cognition, attentional control, semantic priming, subliminal perception

## Abstract

Classical theories assume that unconscious automatic processes are autonomous and independent of higher-level cognitive influences. In contrast, we propose that automatic processing depends on a specific configuration of the cognitive system by top-down control. In 2 experiments, we tested the influence of available attentional resources and previously activated task sets on masked semantic priming in a lexical decision task. In Experiment 1, before masked prime presentation, participants were engaged in an easy or hard primary task that differentially afforded attentional resources. Semantic priming was attenuated when the primary task was hard, that is, when only little attentional resources were available. In Experiment 2, a semantic or perceptual induction task differentially modulated subsequent masked semantic priming. Hence, unconscious automatic processing depends on the availability of attentional resources and is susceptible to top-down control.

## Introduction

For some time, it has been widely accepted that automatic processes are autonomous and immune to the influence of higher-level cognitive functions. Specifically, classical theories of automaticity defined automatic processes as unconscious and independent from capacity-limited resources. Furthermore, automatic processes act in parallel and are not prone to interference with other processes (Posner & Snyder, 1975; Schneider & Shiffrin, 1977). In contrast, controlled processes are, according to these theories, characterized as conscious and are influenced by top-down factors such as attention, task sets and action goals. Consequently, cognitive control can only operate on conscious cognition, while, coincidentally, unconscious automatic processes act unconstrained. This unimpeded occurrence of unconscious processes could cause increased demand for cognitive control and reconfiguration, if the results of automatic processes interfere with the current conscious action plan.

Such an inflexible system, as assumed by classical theories, appeared implausible given research findings about the flexibility and adaptability of the human brain and cognition: Recent studies suggest that top-down factors like attention and intention modulate automatic processes in a context-dependent manner. Therefore, refined conceptualizations of automaticity were proposed (Kiefer, 2007; Naccache, Blandin, & Dehaene, 2002; Neumann, 1984). According to these theories, automatic processes are assumed to be contingent on the configuration of the cognitive system. The term *conditional automaticity* was therefore formed (Bargh, 1989; Logan, 1989).

To investigate automatic processes in isolation, the masked priming paradigm has proven to be an ideal tool. Here, the facilitating effect of an unconsciously presented masked stimulus on the processing of a subsequent visible target is measured. Processing of such a masked stimulus is thought to occur automatically without contribution of strategic influences. Consciously perceived stimuli also trigger automatic processes (Hommel, 2000), however, most likely, controlled processes also contribute (Jacoby, 1991; Koivisto, 1998).

Depending on the relationship between prime and target, different forms of priming can be distinguished. Response priming occurs in two alternative-forced choice RT (reaction time) experiments when prime and target indicate the same motor response. This effect is caused by automatic response preparation processes elicited by the unconsciously perceived prime, which facilitate same-hand responses towards the target (Dehaene et al., 1998; Klotz & Neumann, 1999; Neumann & Klotz, 1994; Verleger, Jaśkowski, Aydemir, van der Lubbe, & Groen, 2004; Vorberg, Mattler, Heinecke, Schmidt, & Schwarzbach, 2003).

Semantic priming refers instead to the facilitated classification of a target word when a preceding prime word is semantically related to the target (Neely, 1991). In contrast to the response priming paradigm, here, prime and target across different relatedness conditions require the same response. Even masked primes elicit semantic priming effects, which have been taken as evidence that the semantic meaning of the prime is unconsciously accessed and automatically pre-activates the semantic target representation (Carr & Dagenbach, 1990; Kiefer, 2002).

 Researchers who have investigated automatic processes by using masked priming paradigms have challenged the classical assumptions by showing that top-down factors influenced masked priming effects and formulated refined concepts of automaticity. Neumann (1984) developed the theory of direct parameter specification (DPS) to explain unconscious response priming. According to the theory, masked primes are only processed and do influence the response to a target if they match current intentions. More generally speaking, unconsciously registered information is used to specify an open parameter of the currently active action plan, thereby triggering a prepared response. Converging evidence for this assumption comes from several studies, which showed that unconscious response priming only occurred when primes were task-relevant and congruent with currently active action-goals (Ansorge, Heumann, & Scharlau, 2002; Ansorge & Neumann, 2005, Eckstein & Perrig, 2007; Kunde, Kiesel, & Hoffmann, 2003). 

Recent studies have shown, additionally, that DPS theory can explain not only unconscious response priming but also subliminal priming of cognitive operations (Mattler, 2003) and subliminal priming of attention (Ansorge, Kiss, & Eimer, in press; Scharlau & Ansorge, 2003). However, DPS theory has no neuro-functional grounding, while the gating framework for unconscious cognition, stated by our research group, accounts for broader variety of cognitive processing and has a neurobiologically plausible basis (Kiefer, 2007). Specifically, we propose that, in unconscious cognition, the parameter specification, or generally speaking, the configuration of the cognitive system, by attention, intention, and task sets, is achieved by a similar kind of gating mechanism as suggested for conscious perception (Hamker, 2005; Kiefer, 2007; Müller, Reimann, & Krummenacher, 2003). Relevant task information is held in dorsolateral prefrontal areas of the brain, while the corresponding information-processing areas are located in posterior regions of the brain. However, both are linked through neural connections. The gating mechanism enhances processing of task-relevant stimulus information while attenuating task-irrelevant information. In neural networks, this mechanism is modelled by increasing the “gain” of neurons in brain areas that process task-relevant stimulus information while decreasing the gain of neurons in other areas (e.g., Cohen & Servan-Schreiber, 1992; Hamker, 2005). The gain is a parameter that increases (high gain) or decreases (low gain) the likelihood that a neuron, at a given activation level, fires. For example, by regulating the gain of sensory neurons, prefrontal areas could enhance sensory processing of task-relevant stimulus features and attenuate the processing of task-irrelevant information. Accordingly, in a masked priming paradigm, unconsciously perceived stimuli can only trigger specific automatic processes (e.g., semantic priming) if the current task representation in prefrontal cortex enhances the corresponding information processing pathway in posterior (semantic) brain areas. However, if the gating mechanism emphasizes other processing pathways, unconsciously perceived stimuli will not be able to elicit further “automatic” processes.

This postulated top-down gating mechanism accounts for unconscious and conscious cognition. However, top-down control for unconscious processing is only pre-emptive, while for conscious processes, reactive control can be administered additionally. In pre-emptive control, top-down influences are set up in advance of unconscious and conscious stimulus presentation, whereas reactive control refers to higher cognitive influences that are set up in response to ongoing or completed conscious stimulus processing. Hence, top-down control of unconscious cognition must occur implicitly on the grounds of currently activated action goals or outcomes of overt behaviour. Consequently, the possibility of intended and reactive top-down modulation remains to be the most prominent distinguishing feature between controlled and automatic processes. In addition, subliminal information cannot be used for determining further strategic processing steps in a deliberate fashion (Merikle, Joordens, & Stolz, 1995). For that reason, conscious “strategic” stimulus processing allows for a greater adaptability and flexibility of top-down control than unconscious “automatic” processing although both forms of processes share basic principles of top-down modulation.

 These refined assumptions about the functional mechanisms of unconscious perception and its susceptibility to top-down control receive support from several studies, which have demonstrated top-down influences on unconscious response and semantic priming. In the context of DPS theory, we have already discussed the necessity of congruence between currently active intentions and masked primes to obtain facilitating response effects (Ansorge et al., 2002; Eckstein & Perrig, 2007; Kunde et al., 2003). In addition to intentions, the dependence of unconscious processes on temporal attention has been demonstrated (Kiefer & Brendel, 2006; Naccache et al., 2002). Kiefer and Brendel (2006), for example, presented an attentional cue in the time window of masked prime presentation in a semantic priming paradigm or already one second earlier. This experimental manipulation prompted the participants’ temporal attention to the masked prime in the short cue prime interval (CPI), but they disengaged temporal attention from the unconsciously presented prime in the long CPI condition. Electrophysiological masked semantic priming effects were only present when the prime appeared in the attended time window. In a similar response priming study (Naccache et al., 2002), masked priming effects were only obtained when the onset of the prime-target pair was temporally predictable and, therefore, attended to. These results suggest that temporal attention is a prerequisite for unconscious priming. Top-down control processes can suppress the impact of misguiding masked primes: Masked response priming effects were considerably reduced when the unconsciously presented prime was incompatible with the target in 80% of the trials, producing erroneous reactions (Jaśkowski, Skalska, & Verleger, 2003; Wolbers et al., 2006). Although being not aware of the masked prime, participants perceived consciously the errors they made. Thus, top-down control was reactively engaged in response to the errors and suppressed interfering subliminal information. 

In the following study, we present two behavioural experiments, in which we tested the influence of available attentional resources (Experiment 1) and previously activated task sets (Experiment 2) on masked semantic priming in a lexical decision task. In Experiment 1, before masked prime presentation, participants were engaged in an easy or hard primary task that differentially afforded attentional resources. In Experiment 2, a semantic or perceptual task set was induced prior to unconscious semantic priming. We expected that both the availability of attentional resources, as well as the currently active task set, would influence subsequent unconscious prime processing.

## Experiment 1

 Following the study of Kiefer and Brendel (2006), in which semantic priming was modulated by an attentional cue, we assume that unconscious automatic processes depend on capacity-limited attentional resources. Specifically, the gating framework (Kiefer, 2007) predicts that further semantic processing of subliminal stimuli requires an attentional amplification of the unconscious stimulus representation. In order to test this assumption, we used two primary tasks that differed significantly in difficulty and had to be performed prior to a semantic masked priming procedure. As the primary tasks differentially drew on processing capacity, available attentional resources should be differentially reduced for a period of several hundred milliseconds following task completion (for a review, see Pashler, Johnston, & Ruthruff, 2001). In the easy primary task, participants had to decide whether or not a presented word contained a capital letter (at any position). When having given the response – 200, 500, 800, or 1100 ms – response-prime-interval (RPI), later a masked prime word, was presented and, subsequently, a target word that afforded a lexical decision: Participants had to decide whether or not the target formed a real word. In the cases in which the masked prime word and the target word were semantically related, we assumed faster lexical decisions towards the target compared with unrelated prime-target pairings (semantic priming effect). In the other half of the trials, participants were engaged in a hard primary task prior to the lexical decision task. Participants had to decide whether the presented word contained a letter at the first or last position with a closed or open shape. If attentional processing capacity is a prerequisite for automatic processing to occur, then masked semantic priming should be larger following the easy, rather than the hard, primary task.

### Methods

#### Participants

Thirty-two healthy, right-handed, native German speakers with normal or corrected-to-normal vision contributed data to this experiment. The data of one participant had to be excluded from analysis, because the identification rate of this participant exceeded the confidence interval of chance performance in the masked prime identification test (more than 65% correct responses). The remaining 31 participants (17 men and 14 women) were in the age range of 17 to 32 years, with a mean of 24 years. Handedness was assessed using a translated version of the *Edinburgh Handedness Inventory* (Oldfield, 1971). All participants signed a written consent form after the nature and the consequences of the experiment had been explained. The experiment was conducted in accordance with the Declaration of Helsinki.

#### Material

For the easy primary task, we presented 160 German words, half of which contained a capital letter at a random position within the word. The other half were written with small letters only. Word length of all words used in the primary tasks ranged from four to seven letters. Participants had to decide as quickly as possible whether or not the displayed word contained a capital letter. Half of the words started or finished with a letter containing at least one closed shape (e.g., A, B, e, g) and the other half started and finished with a letter that contained only open shapes (e.g., E, F, s, u), which served as stimuli in the hard primary task. Here, participants had to decide whether the first or last letter of the presented word contained an open or at least one closed shape. Responses were given by pressing one of the assigned keys with the index or middle finger of the right hand. In a pilot study with 8 participants, reaction times of the performance of these two tasks were assessed. Task order was counterbalanced across participants. Reaction times were, indeed, significantly faster when performing the capital letter search than when making the closed vs. open shape decision (mean RT: 506 vs. 626 ms, *p* < .0001). Error rates were 4.9% for both tasks.

The set of primes and targets for the lexical decision task consisted of 320 German word–word and 320 word–pseudoword pairs, which has been used in earlier priming studies (Kiefer, 2002; Kiefer & Spitzer, 2000). Primes and targets were, on average, five letters long (range three–nine) and subtended at a viewing distance of 90 cm and a visual angle of about 2.6° in width and 0.9° in height. The word–pseudoword pairs served as distracters and were not analysed further. The word–word combinations consisted of 160 semantically related pairs (e.g., ‘‘hen–egg’’) and 160 semantically unrelated pairs (e.g., ‘‘car–leaf’’). Critical prime–target combinations were equated in word length and frequency of the primes (Ruoff, 1990), as well as those of the targets across conditions (pseudowords were only matched in length). Prime-target combinations were divided into eight lists. The assignment of each list to a given experimental condition (combination of primary task and RPI) was counterbalanced across participants. Each participant received different combinations of primary word and prime-target pairings.

#### Procedure

**Figure 1.**
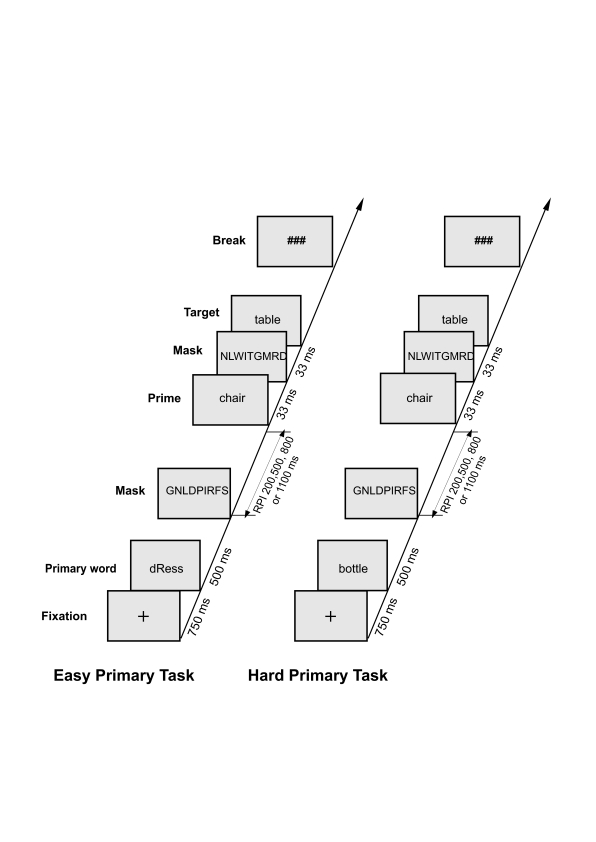
Sequence of events in the experimental paradigm used in Experiment 1, consisting of an easy or hard primary task and subsequent masked semantic priming.

 The total number of 640 trials was divided into eight blocks of 80 trials each. Breaks were provided between the blocks. Figure 1 displays the sequence of events used in the experimental paradigm. In each trial, participants were first presented with a fixation cross for 750 ms, which was followed by a word for 500 ms that represented the stimulus for the primary tasks. Participants had to decide (a) in the easy primary task, whether or not the word contained a capital letter, and (b) in the hard primary task, whether the first or last letter of the word contained an open or a closed shape. As soon as the response was given, a random letter string (forward mask) consisting of 10 capital letters was presented for 200, 500, 800, or 1100 ms (RPI). In either case, the random letter string was followed by the prime word, which was shown for 33 ms. After prime presentation, another random letter string was presented for 33 ms, which served as a backward mask. Thereafter, the target stimulus that either formed a real word or a pronounceable pseudoword was displayed. Participants had to decide as fast and as accurately as possible whether or not the target was a real word. Responses were indicated by pressing one of two buttons with the right index and middle finger. Participants were not informed of the presence of the prime. The target remained on the screen until a response was given. Thereafter, three hash marks were presented, which prompted the participant to initiate the next trial by pressing a button.

All stimuli were displayed in white font against a black background on a computer monitor synchronous with the screen refresh (refresh rate = 16.67 ms). Trial order within each block was randomized, whereas the different primary tasks were presented in blocks. After the priming experiment, participants were informed of the presence of the prime behind the mask and were questioned as to whether they had recognized that prime words had been presented. None of the participants reported awareness of the primes. An objective measure of prime identification was obtained thereafter within a paradigm, which included the same sequence of events as the masked priming paradigm (for details, see Kiefer, 2002). In a visual discrimination task, masked stimuli consisted of 80 words and 80 letter strings. Each letter string comprised nine repetitions of the identical capital letter (e.g., “AAAAAAAAA”), which was randomly selected in each trial. Masked words were either semantically related or unrelated to a subsequently presented unmasked context word (40 trials of each condition). This context word, for which no response was required, was included in order to keep the sequence of events identical to the priming paradigm and to test whether backward priming from the target to the masked prime had occurred. Stimulation parameters were identical to the main experiment. The only difference was that only the RPI condition with 1100 ms was realized. This condition should provide a liberal estimation of masked prime identification for the shorter RPIs, because at the longest RPI, the masking influence of the primary task word is reduced. Participants were instructed to perform the easy or hard primary task on the first visible word. Thereafter, their task was to decide whether the masked stimulus was a word or a letter string. Instructions stressed accuracy over response speed. Participants were also requested to make their best guess when they did not feel confident about the correct response.

### Results

#### Masked word identification test

 We assessed the visibility of the masked primes in an identification test following the priming phase. As noted above, data of one participant had to be excluded from further analysis because identification rate of this participant exceeded the confidence interval of chance performance. For the remaining 31 participants, identification performance was distributed around the chance level of 50% (mean easy = 48.8%, mean hard = 52.4%), which is expected by mere guessing. In order to assess whether the targets facilitated identification of related masked primes (backward priming), *d*’ sensitivity measures for the semantically related and unrelated conditions were calculated from each participant’s hit rates (correct responses to words) and false alarm rates (erroneous responses to letter strings) according to Green and Swets (1966). The measure *d*’ reflects whether the hit/false alarm rate distributions of related prime-target pairs and unrelated prime-target pairs are identical (*d*’ = 0) or have no overlaps. A repeated-measures analysis of variance (ANOVA) on *d*’ measures with the within-subject factors semantic relatedness and task difficulty revealed a main effect for task difficulty, *F*(1, 30) = 8.7, *p* = .01, reflecting a somewhat lager visibility of the masked prime, when the hard primary task was performed before masked prime presentation, *d*’ = 0.14 (hard) vs. *d*’= -0.08 (easy). However, no interaction with semantic relatedness was observed, *F*(1, 30) = 0.1, *p* = .80, which excludes that backward priming rendered the masked prime words partially recognizable. An additional t-test was performed to test whether *d*’ differed significantly from zero (i.e., chance performance). For the easy primary task, *d*’ did not differ from chance performance, *t*(30) = -1.3, *p* = .21, whereas for the hard primary task, *d*’ was significantly larger than zero, *t*(30) = 2.5, *p* = .018. That said, the value of *d*’ = 0.14 is very small, suggesting that participants were extracting no or only little information from the masked prime.

#### Primary task to manipulate availability of attentional resources

Of all response times to the primary tasks, the slowest 15% of trials^1^ of each subject were rejected as outliers. Separate ANOVAs with repeated-measures were calculated on median reaction time (RT) and error rate (ER) that included the factor primary task (easy vs. hard). Responses were significantly faster in the easy than in the hard primary task, 662 vs. 835 ms, *F*(1, 30) = 73.2, *p* < .0001. An identical analysis of the error rate revealed a similar pattern. Performance was significantly less error prone in the easy than in the hard primary task, 1.9% vs. 3.8%, *F*(1, 30) = 26.9, *p* < .0001.

#### Masked priming

**Figure 2.**
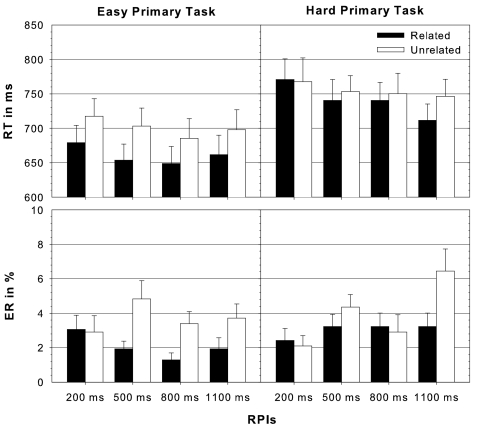
Median and standard error of reaction times (RT, upper panel) and error rates (ER, lower panel) in the lexical decision task towards semantically related (related - black) and unrelated (unrelated - white) prime-target pairings under easy and hard primary task conditions, respectively, and separately for each response-prime-interval (RPI = 200, 500, 800, and 1100 ms).

Of all response times to the lexical decision task, the slowest 15% of trials of each subject were defined as outliers. This resulted in the removal of 6.8% of trials from the relevant dataset (word-word pairings). ANOVAs with repeated-measures on the factors primary task difficulty, and RPI and semantic relatedness were performed on median RT and ER. For the RT data all three main effects were significant. Lexical decisions were much faster when the previous primary task was easy rather than hard, *F*(1, 30) = 32.8, *p* < .0001; 681 vs. 748 ms. The RPI influenced response times towards the target significantly in the way that with increasing RPI response time decreased, *F*(3, 90) = 7.7, *p* < .001; 734 vs. 713 vs. 706 vs. 705 ms. Importantly, the semantically related prime-target pairs facilitated significantly the lexical decision towards the target compared with semantically unrelated pairings, *F*(1, 30) = 19.2, *p* < .0001; 701 vs. 728 ms. This effect was further qualified by the two-way interaction of primary task difficulty by semantic relatedness, *F*(1, 30) = 8.7, *p* = .0062. Following the easy primary task, masked priming effects were much larger, *F*(1, 30) = 39.0, *p* < .0001, Δ*m* = 40.2 ms, compared with priming effects following the hard primary task, Δ*m* = 13.5 ms (see Figure 2). In fact, masked priming in the hard primary task condition was not significant, *F*(1, 30) = 2.5, *p* = .13. Figure 2 illustrated the reaction time data and error rates separately for both primary task and the different RPIs.

**Figure 3.**
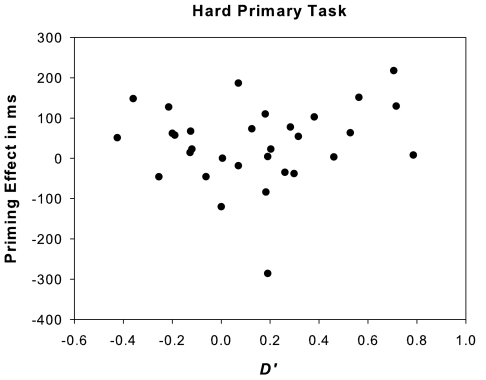
Correlation between the individual *d*’ value for masked prime recognition following the hard primary task (x axis) and the corresponding priming effect (in milliseconds) of the lexical decision task following the hard primary task and a 1100 ms RPI (y axis: median RT to semantically unrelated prime-target pairings minus RT to related prime target pairings).

As the *d*’ prime identification measure was significantly larger than zero following the hard primary task, we calculated for this primary task condition the correlation between the individual *d*’ and priming effect. This analysis was performed in order to determine a possible relationship between prime identification performance and masked priming effects. We only assessed priming effects at the 1100 ms RPI, because this RPI was used in the prime identification test, from which *d*’ measures were derived. As one can see in Figure 3, there was no correlation between masked prime recognizability and the priming effect (*r* = .14, *p* > .47), ruling out a contribution of conscious stimulus identification to masked priming.

When performing an identical ANOVA on ER, a main effect for semantic relatedness was obtained, *F*(1, 30) = 10.7, *p* = .0027. Participants committed significantly fewer errors when the target was preceded by a semantically related prime than when the prime had no semantic relation to the target( 3.2% and 4.4%, respectively). This effect was further qualified by the two-way interaction RPI by semantic relatedness, *F*(3, 90) = 2.7, *p* = .05. Planned contrast revealed significant priming effects in the 500 and 1100 ms RPI, but not in the other two RPIs, *F*s > 7.2, *p*s < .012 vs. *F*s < 1.8, *p*s > .19, respectively (see Figure 2). Primary task difficulty showed no effect at all on the error rates, that is, the three-way interaction was not significant, *F* (3, 90) = 1.9, *p* = .16.

### Discussion

The major aim of this experiment was to investigate whether the availability of attentional processing capacity affects unconscious information processing. Specifically, we analysed the dependency of masked semantic priming effects on the cognitive demands of a previously performed task. First of all, as predicted, masked semantic priming was significantly reduced following a hard primary task in comparison with an easy one. Hence, subliminal processing, crucially, depends on the availability of attentional resources: A reduction of attentional resources in the time window of masked prime presentation attenuates priming effects. The present results are clearly incompatible with classical theories of automaticity assuming independence of automatic processes from capacity-limited attention (Posner & Snyder, 1975; Schneider & Shiffrin, 1977). The present experiment, therefore, confirms and extends earlier studies on the attentional modulation of unconscious processing. Our results are in line with earlier demonstrations of the influence of temporal attention on masked priming (Kiefer & Brendel, 2006; Naccache et al., 2002). In extending this line of research, we showed for the first time that unconscious processing depends on the availability of attentional capacity.

A closer look at the magnitude of priming effects as a function of RPI is suggestive of a differential priming pattern following the easy and hard primary tasks. Following the easy primary task, semantic priming effects were significant for all RPI conditions and also exhibited a quite comparable magnitude, *F*s > 9.4, *p*s < .0045, Δ*m* >36.5 ms. Intriguingly, following the hard primary task, semantic priming was entirely blocked in the shorter RPIs (*F*s < 1, Δ*m* < 12.8 ms) but recovered when there was sufficient time (1100 ms) between the completion of the primary task and masked prime presentation, *F*(1, 30) = 3.9, *p* = .057, Δ*m* = 35 ms. This pattern of masked priming effects on the different primary task conditions signals that, as outlined above, attentional capacity plays an important role in the processing of unconsciously presented stimuli. However, this differential priming pattern could, additionally, reflect the influences of different task sets on masked priming. The primary tasks did not only differ with regard to their difficulty, but also with regard to how the word stimulus had to be processed. For the hard task, only the first and the last letters of the word were task relevant, whereas the easy task required scanning the whole word to search for a capital letter that could be at any position within the presented word. Thus, the primary tasks could have induced two different task sets, which had been implicitly applied to the masked prime: In the hard task, it was required to attend to perceptual letter features and to ignore the entire word form. This perceptual task set could have still been active in the first hundred milliseconds after task completion, thereby attenuating semantic processing of the prime word at the shorter RPIs. In contrast, for the easy primary task, the word stimuli had to be attended to as a whole because the capital letter appeared at a random location within the word. Accordingly, the easy primary task could have induced a task set that includes attention to the entire word and implicit word reading (Brass, Derrfuss, & von Cramon, 2005; Cohen, Dunbar, & McClelland, 1990). As a result, if this task set had been implicitly applied to the masked prime, the subliminally presented word was semantically processed at any RPI. Hence, the pattern of priming effects could reflect the modulatory effects of task sets on unconscious semantic priming, in addition to the clear top-down influences of attentional capacity. To investigate possible task set effects on subsequent masked semantic priming, we designed a new experiment that used primary tasks of a comparable level of difficulty, which were expected to induce different forms of task sets. Consequently, we will refer to these primary tasks in Experiment 2 as induction tasks.

## Experiment 2

 In this second experiment, we explored the modulatory effects of *task sets* on masked semantic priming. Task sets are defined as adaptive configurations of the cognitive system for efficient performance in a given task (Gilbert & Shallice, 2002; Rogers & Monsell, 1995). This task-dependent configuration persists for a while, even after task completion, an effect that is known as task set inertia (Allport, Styles, & Hsieh, 1994). Hence, according to the gating framework of top-down control of unconscious cognition, task sets should be able to influence subsequent subliminal priming. To investigate the influences of task sets on masked priming, participants were engaged into a semantic or perceptual induction task that should activate either a semantic or a perceptual task set. After having given the response to the stimulus of the induction task, they underwent a masked semantic priming procedure. According to the gating framework, this activated task set should modulate masked priming effects. In detail, the induction task required either a semantic word categorization (living vs. non-living object), or a perceptual word categorization, the same as the hard primary task in Experiment 1, that is, first or last letter with closed or open shape. Subsequently, a masked prime word was displayed and followed by a lexical decision to the target. Combining knowledge about task-switching and task configuration processes with our proposed gating framework, we infer a specific temporal dependency of modulatory effects of task sets on masked semantic priming. The time course of the reconfiguration process in task-switch conditions was accessed by Rogers and Monsell (1995). Specifically, they investigated the influence of five response-stimulus intervals on shift costs using the alternating runs paradigm, in which the task switch was predictable. Their results indicated that the reconfiguration process for a change of task lasts for approximately 600 ms. Furthermore, there is evidence of active inhibition of task sets when the task has been completed (Mayr & Keele, 2000). Thus, the time interval between response to the induction task and presentation of the subliminal prime (RPI) could be of importance for modulatory task-set influences on semantic priming effects. 

In order to assess these temporal dynamics of top-down modulation in detail, we systematically manipulated the RPI as in Experiment 1. With 200, 500, 800, and 1100 ms, we chose equidistant RPIs in order to see whether the modulatory task set effects on semantic priming were gradual or more of an all-or-nothing pattern. When performing the semantic induction task, a corresponding task set will be activated and semantic processing pathways will be emphasized for around 600 ms. As a consequence, semantic processing of the subsequently presented masked prime will be facilitated within this time window. Hence, we expect to observe a robust priming effect to targets in the lexical task when the masked prime is presented shortly after the response to the induction task (RPI = 200 and 500 ms). However, when performing a perceptual induction task, the configuration of the cognitive system will emphasize perceptual processing of the subsequently presented masked prime. No, or only minimal, semantic information can be retrieved from the prime at a short RPI, which attenuates semantic priming in the following lexical decision task. However, according to the study by Rogers and Monsell (1995), the task set evoked by the induction task should have decayed or been actively suppressed (Mayr & Keele, 2000) when the masked prime is presented at a time point later than 600 ms after the response to the induction task. Consequently, if the RPI between the perceptual induction task and the following masked semantic prime is large enough (800 and 1100 ms), the emphasis on perceptual processing diminishes. This should allow for semantic processing of the masked prime and result in a semantic priming effect. The opposite effect should be observed for a long RPI after the semantic induction task: At this long RPI, the semantic task set should be suppressed (Mayr & Keele, 2000) so that semantic processing of the masked prime would be abolished. As a consequence, we expect a reduction of semantic priming for a semantic induction task after a long RPI. 

### Methods

#### Participants

Forty-one, right-handed (Oldfield, 1971), native German speakers with normal or corrected-to-normal vision contributed data to this experiment. In total, 10 participants had to be excluded, 6 because the identification rate of these participants exceeded the confidence interval of chance performance in the masked prime identification test and 4 due to high error rates and/or too many outliers. The remaining 31 participants (18 men, 13 women) were in the age range of 20 to 44 years, with a mean of 24.6 years. All participants signed a written consent form after the nature and the consequences of the experiment had been explained. The experiment was conducted in accordance with the Declaration of Helsinki.

#### Material and procedure

The stimulus sets for primes and targets, the timing of all events as well as their analysis were identical to Experiment 1. The only difference pertained to the primary tasks. While in Experiment 1 primary task difficulty was manipulated, this experiment aimed to investigate the differential effect of a perceptual and a semantic task set. In order to do so, we chose the hard primary task (closed vs. open-letter shape) from Experiment 1 as a task to induce a perceptual task set (perceptual induction task) and created a second induction task that should activate a semantic task set. We used 160 German words, therefore, half of which described living objects (e.g., “pilot”, “apple”, “dog”) and the other half referred to non-living objects (e.g., “castle”, “pencil”, “bottle”), as word stimuli for the semantic task. Word length of all stimuli of the induction tasks ranged from five to six letters and they were equated for word frequency. This stimulus set was tested in a pilot experiment. Fifteen participants (9 men and 6 women) with an average of 22.4 years performed the induction tasks in separate blocks. Task order was counterbalanced across participants. The perceptual task required participants to decide whether the first or last letter of the presented word contained an open or a closed shape. In the semantic task, participants decided whether the presented word described a living or a nonliving object. Responses were given by pressing one of the assigned keys with the index or middle finger of the right hand. Median response times of correct answers and error rates did not show a significant difference between the perceptual and the semantic task, 720 vs. 754 ms, *p* = .23, and 3.6% vs. 6.9%, *p* = .13 respectively.

Besides the different induction tasks, all the other experimental parameters, including the recognition test, were identical with those in Experiment 1 (see Figure 1).

### Results

#### Masked word identification test

We assessed the visibility of the masked primes in an identification test following the priming phase. As noted above, data of 6 participants had to be excluded from further analysis because identification rate of these participants exceeded the confidence interval of chance performance or because they reported having recognized the masked prime. For the remaining 31 participants, identification performance was distributed around the chance level of 50% (mean perceptual = 48.7% and semantic = 49.1%), which is expected by mere guessing. Repeated-measures ANOVA on *d*’ measures (for details, see Experiment 1) with the within-subject factors semantic relatedness and induction task revealed no significant differences between conditions, *F*s < 2.4, *p*s > .128, which excludes that backward priming rendered the masked prime words partially recognizable. Additional *t*-tests of *d*’ against zero show no significant difference from chance performance, neither after the semantic induction task; *d*’ = - 0.13, *t*(30) = -1.8, *p* = .081; nor after the perceptual induction task, *d*’ = - 0.03, *t*(30) = -.4, *p* = .68.

#### Induction task to activate task sets

Of all response times to the induction tasks, the slowest 15% of trials of each subject were defined as outliers. Repeated-measures ANOVAs on median reaction time (RT) and error rate (ER) with the within-subject factor induction task was performed. Semantic decisions were made significantly faster than perceptual decisions, 772 vs. 820 ms, *F*(1, 30) = 12.6, *p* = .002. An identical analysis of the error rates revealed a reverse pattern. Participants produced significantly more errors in the semantic induction task than in the perceptual one, 11% vs. 5.5%, *F*(1, 30) = 28.4, *p* < .0001.

#### Masked priming

**Figure 4.**
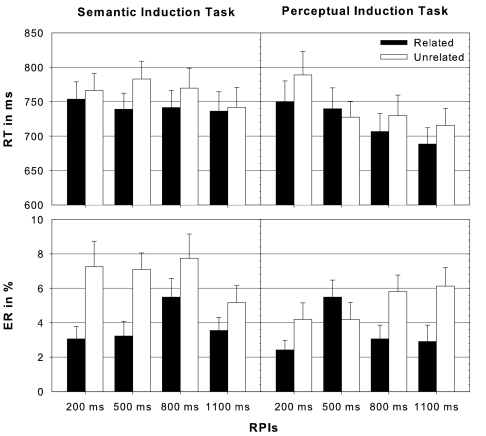
Median and standard error of reaction times (RT, upper panel) and error rates (ER, lower panel) in the lexical decision task towards semantically related (related - black) and unrelated (unrelated - white) prime-target pairings under semantic and perceptual induction task conditions respectively and separately for each response-prime-interval (RPI = 200, 500, 800, and 1100 ms).

Of all response times to the lexical decision task, the slowest 15% of trials of each subject were rejected as outliers. This resulted in the removal of 7.5% of trials from the relevant dataset (word-word pairings). Repeated-measures ANOVAs on median RT and ER with the within-subject factors induction task, RPI, and semantic relatedness were performed. RT and ER results are displayed in Figure 4. All three main effects were significant. Lexical decisions were faster, when the previously activated task set was perceptual rather than semantic, *F*(1, 30) = 7.1, *p* = .013, 731 vs. 754 ms. The RPI influenced response times towards the target significantly in that with increasing RPI, response time decreased, *F*(3, 90) = 10.6, *p* < .0001, 765 vs. 747 vs. 737 vs. 721 ms. Most importantly, semantically related prime-target pairs facilitated significantly the lexical decision towards the target, compared with semantically unrelated pairings; *F*(1, 30) = 15.8, *p* < .001, 732 vs. 753 ms; an effect that was further qualified by the three-way interaction of induction task by RPI by semantic relatedness, *F*(3, 90) = 2.8, *p* = .045. Planned contrasts, comparing response times with semantically related and unrelated prime-target pairs separately for each induction task and RPI condition, revealed an opposite pattern of priming effects for a previously induced semantic and perceptual task set respectively, dependent on the RPI. Unexpectedly, for the 200 ms RPI, no priming effect was observed when a semantic task set was induced, *F*(1, 30) = 1.0, *p* = .32, Δ*m* = 12.2 ms. But, when a perceptual task set was induced, lexical decisions towards target words were significantly facilitated by semantically related primes, an effect that was not observed in the identical task and RPI in Experiment 1, *F*(1, 30) = 5.8, *p* = .023, Δ*m* = 38.4 ms. This pattern was reversed for the 500 ms RPI. Here, an induced semantic task set yielded a significant masked priming effect; *F*(1, 30) = 14.9, *p* < .001, Δ*m* = 43.7 ms; whereas an induced perceptual task set prevented masked priming, *F*(1, 30) = 0.5, *p* = .47, Δ*m* = -12.2 ms. A significant priming effect was not observed under any induction task condition for either the 800 or for the 1100 ms RPI. However, the quantitative pattern, as can be seen in Figure 4, indicated increased priming for preceding perceptual task set induction; Δ*m* = 23.3 ms (800 ms RPI) vs. 27.1 ms (1100 ms RPI); and decreased priming for preceding semantic task set induction, Δ*m* = 28.1 ms (800 ms RPI) vs. 5.4 ms (1100 ms RPI).

An equivalent ANOVA performed on the error rates showed similar effects to the RT data. The main effects induction task and semantic relatedness were significant. Lexical decisions were more error prone when previously a semantic task set was induced than a perceptual one, *F*(1, 30) = 4.3, *p* = .046, 5.3% vs. 4.3%. Responses to targets that were preceded by a semantically related prime were more often correct than when preceded by a semantically unrelated word, 3.6% vs. 5.9%, *F*(1, 30) = 27.8, *p* < .0001. This effect was further qualified by the predicted three-way interaction induction task by RPI by semantic relatedness, *F*(3, 90) = 3.3, *p* = .0233. Planned contrast revealed a clear pattern of significant priming effects for the 200 and 500 ms RPI subsequent to a semantic induction task; *F*(1, 30) = 8.2, *p* = .0077 (Δ = 4.2%); and *F*(1, 30) = 11.8, *p* = .0018 (Δ = 3.9%), respectively. No priming effect, however, occurred for the 800 and 1100 ms RPIs; Fs(1, 30) < 2.7, ps > .11; subsequent to a semantic induction task (Δ = 2.3 and 1.6%). However, subsequent to a perceptual induction task, no priming effect was observed for the 200 and 500 ms RPIs; Fs(1, 30) < 3.3, ps > .078 (Δ = 1.8% and -1.3%); but for the 800 and the 1100 ms RPIs; *F*(1, 30) = 7.4, *p* = .011 (Δ = 2.7%); and *F*(1, 30) = 5.8, *p* = .023 (Δ = 3.2%), respectively.

### Discussion

The present results demonstrated a differential modulation of masked semantic priming effects by the induced task set. In detail, we observed a three-way interaction between induction task, semantic relatedness, and RPI in both RT as well as ER. The priming pattern in the ER data was quite straightforward: Semantic priming occurred when a semantic task set was active shortly before the presentation of the masked prime (RPIs of 200 and 500 ms). However, when a perceptual task set was induced, priming effects were abolished at these short RPIs. In the long RPI conditions instead (800 and 1100 ms), semantic priming was absent after the semantic induction task, but priming effects recovered after a perceptual one. While the RT priming effects at RPIs of 500 ms and greater also showed this pattern, they were deviant at the shortest RPI of 200 ms. We would like to refer to a more recent study at this point, in which we used the identical tasks but the double amount of trials because only two RPIs were administered. In this study, we replicated for the perceptual induction task the identical pattern from Experiment 1 (no semantic priming at the 200 ms RPI) and found a reliable semantic priming effect subsequently to the semantic induction task in the 200 ms RPI (Kiefer & Martens, submitted). For that reason, we assume that the limited amount of trials, and the resulting lower signal-to-noise-ratio, is responsible for the unexpected RT priming effects at the shortest RPI in the present experiment.

The present masked priming results as a function of the RPIs are in accordance with the known time course of task configuration during task switching (Rogers & Monsell, 1995). It has been shown that a task set is active for about 600 ms. Thereafter, the task set is deactivated and the cognitive system is being reconfigured to meet the new task demands. In line with these findings in task switching, the semantic induction task opens semantic processing pathways for an interval of several hundred milliseconds (RPI of 200 and 500 ms) and allows for subsequent masked prime processing at a semantic level, resulting in semantic priming effects. However, at longer time intervals (RPIs of 800 and 1100 ms), the semantic task set is no longer active, and semantic processing of the masked prime is attenuated as result of a backward inhibition process, which refers to an inhibition of a task set once the task has been actively completed (Mayr & Keele, 2000). For that reason, when participants have sufficient time to abandon the semantic classification task, the semantic task set is deactivated. This deactivation of a semantic task set takes place even during the concurrent preparation of the lexical decision task, whose task set predominantly comprises lexical processing but only to some extent semantic processing. This interpretation could explain why lexical decisions are slower subsequent to the semantic induction task in comparison to the perceptual induction task, although within the main experiment the semantic induction task was slightly easier to perform and therefore less capacity-demanding. In the pilot study, in which performance of the induction tasks was assessed in isolation, both tasks exhibited a comparable level of difficulty.

The perceptual induction task, instead, emphasizes pathways that are involved in visual letter encoding and attenuates other processes for several hundred milliseconds. The meaning of the masked prime cannot be analysed and no semantic priming can occur. At longer RPIs, however, the backward inhibition process deactivates the perceptual task set, and the cognitive system has time to reconfigure for the lexical decision task. Under this cognitive configuration, semantic processing pathways are opened, and an unconsciously presented prime triggers automatic semantic processes.

## General discussion

The present study investigated the effects of attentional capacity and currently active task sets on unconscious semantic priming. We used an experimental paradigm, in which participants were engaged in two primary tasks that differed in difficulty (Experiment 1) or in a semantic or perceptual induction task (Experiment 2). Subsequently, participants underwent masked semantic priming within a lexical decision task. In Experiment 1, the primary tasks served to manipulate the availability of attentional capacity prior to the presentation of the unconsciously perceived prime word. The effectiveness of this manipulation is demonstrated not only by the performance difference for the primary tasks themselves, but also by the carry-over effects to the subsequent lexical decision task (Pashler et al., 2001): The reduced availability of attentional resources following the hard primary task, compared with the easy primary task, is also reflected in the considerable slowing of lexical decisions. However, most critically to show that we were not just measuring unspecific slowing effects, the masked semantic priming effect was differentially modulated by task difficulty. We showed for the first time that attentional processing capacity is clearly a prerequisite for masked prime processing and the observation of unconscious semantic priming effects. Masked primes led only to the facilitation of the processing of semantically related target words when the preceding primary task was easy and required less cognitive resources compared with the hard primary task. This finding clearly challenges classical theories of automaticity, since these assume that unconscious processes are autonomous and can act in parallel to, and independent from, other cognitive processes. However, refined theories of automaticity, as the gating framework (Kiefer, 2007), actually predict a dependence of unconscious processing on top-down amplification.

In addition to the strong effect of attentional capacity,Experiment 1 was suggestive of the influence of task sets on masked semantic priming. As outlined in the discussion of Experiment 1, the task demands between the easy and hard primary task were different. While the easy task involved attention to the entire word, the hard task required only attention to single-letter features. To explore this possibility further, we conducted Experiment 2, which investigated the differential effect of task sets on subsequent masked semantic priming. We used semantic and perceptual induction tasks with a comparable level of task difficulty according to a pilot study, in order to induce corresponding task sets. In Experiment 2, within the context of the lexical decision task, the semantic induction task was slightly easier than the perceptual induction task, but still, the difference of difficulty was much smaller than in Experiment 1 (Experiment 1: 173 ms, Experiment 2: 48 ms). We reasoned that the task set – semantic or perceptual – activated by the induction tasks, configures the cognitive system of the participant in a specific way for a limited period of time and enhances or attenuates semantic and perceptual processing pathways respectively (Kiefer, 2007). As a consequence, when a masked semantic prime word was presented shortly after the induction task, the activated task set determined whether or not the unconsciously perceived word was processed at a semantic level and elicited priming effects.

Experiment 2 demonstrated that masked semantic priming was indeed differentially influenced by the different previously activated task sets. Previous studies, investigating unmasked (visible) semantic priming found modulatory effects of prime tasks, as reviewed in (for a review, see Maxfield, 1997). Here, semantic priming was reduced or absent when the task required attention to perceptual letter features of visible prime words, for example, a letter search task, and not their semantic analysis (Chiappe, Smith, & Besner, 1996; Mari-Beffa, Valdes, Cullen, Catena, & Houghton, 2005). It is notable that both automatic spreading of activation in the semantic network and controlled conscious strategic processes contribute to the processing of visible primes (Posner & Snyder, 1975). This makes a co-occurrence of automatic and strategic processes most likely (Jacoby, 1991; Koivisto, 1998). Consequently, one has to eliminate conscious prime identification, in order to study solely automatic processing without contamination of strategic processes. We ensured this by masking the prime and measuring its recognizability individually.

Divergent results in the literature led to the debate as to whether or not semantic processing is automatic. Several studies (Carr & Dagenbach, 1990; Kiefer, 2002; Kiefer & Spitzer, 2000; Rolke, Heil, Streb, & Henninghausen, 2001) have demonstrated reliably the facilitation of target processing by semantically related unconsciously perceived primes. As outlined earlier, these findings provide support for automatic semantic processing, since strategic processes cannot contribute to unconscious prime analysis. In contrast, the above-mentioned prime task effects on conscious priming have been taken as support for the view that semantic processing depends on controlled memory retrieval in congruency with attentional task representations (e.g., semantic orientation towards the prime stimulus). Importantly, our demonstration that masked semantic priming can be top-down modulated by the availability of attentional capacity and task sets, suggests that unconscious semantic processing, and the notion of attentional top-down control, is not necessarily a contradiction, as previously thought. Semantic processing can occur automatically, in the sense that it is initiated without deliberate intention, but unconscious “automatic” semantic processing underlies attentional top-down amplification and control, and is only elicited if the cognitive system is configured accordingly. Such a configuration is induced in classical masked priming experiments without a preceding induction task by the preparation for the target task (e.g., a lexical decision or naming task). The attentional orientation towards word recognition, in contrast with perceptual letter identification, opens the pathway for unconscious semantic processing of the masked prime (see also Valdes, Catena, & Mari-Beffa, 2005). Earlier findings of prime task effects do not question, but strongly support, refined theories of automaticity, which stress the necessity for an appropriate top-down configuration of the cognitive system for automatic processes to occur (Dehaene & Naccache, 2001; Kiefer, 2007; Neumann, 1984). In fact, refined theories of automaticity explicitly predict such an interaction between prime task and semantic priming. We therefore argue that the concept of automaticity, which was defined by independence of attentional top-down factors and by autonomy (Posner & Snyder, 1975; Schneider & Shiffrin, 1977), should be replaced by the notion of conditional automaticity (Bargh, 1989).

The results of our experiments are congruent with those of studies on prime task effects, which we discussed earlier, suggesting that conscious and unconscious semantic processes are governed by similar computational principles. This is, in line with the assumption of the gating framework (Kiefer, 2007), since we assume that explicit tasks on visible primes configure the cognitive system in the same way as implicit task sets. Although our results suggest that consciously controlled and unconscious automatic processes underlie similar computational properties, there are certain limitations. One has to distinguish between the different forms of control that can operate in unconscious and conscious processes. Preemptive, top-down influences are set up in advance of stimulus presentation and can be exerted for both conscious and unconscious stimulus presentations. However, reactive control refers to strategic processes that are established in response to ongoing or completed analysis of consciously perceived stimuli (Ansorge & Horstmann, 2007; Kiefer, 2007): Conscious processing, presumably, remains a prerequisite for more specific and flexible strategic control.

The present experiments support the view that unconscious processing depends on attentional capacity and is susceptible to top-down control. Yet, the finely grained mechanisms underlying these attentional effects on subliminal stimulus processing have to be determined. However, we argue that such an implicit top-down control of unconscious automatic processing optimizes the cognitive system for pursuing an intended goal by prioritizing task-congruent information and suppressing interfering influences. Consequently, this mechanism considerably reduces the risk that unintended and not goal-related unconscious processes determine cognition, and eventually influences behaviour.

## Footnotes

1 This criterion was chosen due to the few trials (20 per condition) and high variance in the response times of the lexical decision and also applied to the primary tasks to use comparable analysis parameters. Analyses of the untrimmed data (using all trials) of both experiments showed identical effect patterns as reported for the trimmed data.

## References

[R1] Allport A., Styles E. A., Hsieh S., Umilta C., Moscovitch M. (1994). Shifting intentional set: Exploring the dynamic control of
						tasks.. Attention and performance 15: Conscious and nonconscious information
						processing. Attention and Performance series..

[R2] Ansorge U., Heumann M., Scharlau I. (2002). Influences of visibility, intentions, and probability in a
						peripheral cuing task.. Consciousness and Cognition.

[R3] Ansorge U., Horstmann G. (2007). Preemptive control of attentional capture by colour: Evidence
						from trial-by-trial analyses and orderings of onsets of capture effects in
						reaction time distributions.. Quarterly Journal of Experimental Psychology.

[R4] Ansorge U., Kiss M., Eimer M. Goal-driven attentional capture by invisible colours: Evidence
						from event-related potentials.. Psychonomic Bulletin & Review.

[R5] Ansorge U., Neumann O. (2005). Intentions determine the effect of invisible metacontrast-masked
						primes: Evidence for top-down contingencies in a peripheral cueing
						task.. Journal of Experimental Psychology: Human Perception and
						Performance.

[R6] Bargh J. A., Uleman J. S., Bargh J. A. (1989). Conditional automaticity: Varieties of automatic influence in
						social perception and cognition. Unintended thought.

[R7] Brass M., Derrfuss J., von Cramon D. Y. (2005). The inhibition of imitative and overlearned responses: A
						functional double dissociation.. Neuropsychologia.

[R8] Carr T. H., Dagenbach D. (1990). Semantic priming and repetition priming from masked words:
						Evidence for a center-surround attentional mechanism in perceptual
						recognition.. Journal of Experimental Psychology: Learning, Memory and
						Cognition.

[R9] Chiappe P. R., Smith M. C., Besner D. (1996). Semantic priming in visual word recognition: Activation blocking
						and domains of processing.. Psychonomic Bulletin and Review.

[R10] Cohen J. D., Dunbar K., McClelland J. L. (1990). On the control of automatic processes: A parallel distributed
						processing account of the Stroop Effect.. Psychological Review.

[R11] Cohen J. D., Servan-Schreiber D. (1992). Context, cortex, and dopamine: A connectionist approach to
						behavior and biology in schizophrenia.. Psychological Review.

[R12] Dehaene S., Naccache L. (2001). Towards a cognitive neuroscience of consciousness: Basic evidence
						and a workspace framework.. Cognition.

[R13] Dehaene S., Naccache L., LeClec‘H G., Koechlin E., Mueller M., Dehaene-Lambertz G. (1998). Imaging unconscious semantic priming.. Nature.

[R14] Eckstein D., Perrig W. J. (2007). The influence of intention on masked priming: A study with
						semantic classification of words.. Cognition.

[R15] Gilbert S. J., Shallice T. (2002). Task switching: A PDP model.. Cognitive Psychology.

[R16] Green D. M., Swets J. A. (1966). Signal detection theory and psychophysics..

[R17] Hamker F. H. (2005). The reentry hypothesis: The putative interaction of the frontal
						eye field, ventrolateral prefrontal cortex, and areas V4, IT for attention
						and eye movement.. Cerebral Cortex.

[R18] Hommel B., Monsell S., Driver J. (2000). The prepared reflex: Automaticity and control in
						stimulus-response translation.. Attention and performance 18: Control of cognitive processes. Attention
						and performance series..

[R19] Jacoby L. L. (1991). A process dissociation framework: Separating automatic from
						intentional uses of memory.. Journal of Memory & Language.

[R20] Jaśkowski P., Skalska B., Verleger R. (2003). How the self controls its “automatic pilot”
						when processing subliminal information.. Journal of Cognitive Neuroscience.

[R21] Kiefer M. (2002). The N400 is modulated by unconsciously perceived masked words:
						Further evidence for an automatic spreading activation account of N400
						priming effects.. Cognitive Brain Research.

[R22] Kiefer M. (2007). Top-down modulation of unconscious
						‘automatic’ processes: A gating
						framework.. Advances in Cognitive Psychology.

[R23] Kiefer M., Brendel D. (2006). Attentional modulation of unconscious
						‘automatic’ processes: Evidence from event-related
						potentials in a masked priming paradigm.. Journal of Cognitive Neuroscience.

[R24] Kiefer M., Martens U. Attentional sensitization of unconscious cognition: Task sets
						modulate subsequent masked semantic priming..

[R25] Kiefer M., Spitzer M. (2000). Time course of conscious and unconscious semantic brain
						activation.. NeuroReport.

[R26] Klotz W., Neumann O. (1999). Motor activation without conscious discrimination in metacontrast
						masking.. Journal of Experimental Psychology: Human Perception and
						Performance.

[R27] Koivisto M. (1998). Categorical priming in the cerebral hemispheres: Automatic in the
						left hemisphere, postlexical in the right hemisphere?. Neuropsychologia.

[R28] Kunde W., Kiesel A., Hoffmann J. (2003). Conscious control over the content of unconscious
						cognition.. Cognition.

[R29] Logan G. D., Bargh U. J. S., Bargh J. A. (1989). Automaticity and cognitive control.. Unintended thought.

[R30] Mari-Beffa P., Valdes B., Cullen D. J., Catena A., Houghton G. (2005). ERP analyses of task effects on semantic processing from
						words.. Cognitive Brain Research.

[R31] Mattler U. (2003). Priming of mental operations by masked stimuli.. Perception & Psychophysics.

[R32] Maxfield L. (1997). Attention and semantic priming: A review of prime task
						effects.. Consciousness & Cognition.

[R33] Mayr U., Keele S. W. (2000). Changing internal constraints on action: The role of backward
						inhibition.. Journal of Experimental Psychology: General.

[R34] Merikle P. M., Joordens S., Stolz J. A. (1995). Measuring the relative magnitude of unconscious
						influences.. Consciousness and Cognition.

[R35] Müller H. J., Reimann B., Krummenacher J. (2003). Visual search for singleton feature targets across dimensions:
						Stimulus- and expectancy-driven effects in dimensional
						weighting.. Journal of Experimental Psychology: Human Perception &
						Performance.

[R36] Naccache L., Blandin E., Dehaene S. (2002). Unconscious masked priming depends on temporal
						attention.. Psychological Science.

[R37] Neely J. H., Besner D., Humphreys G. W. (1991). Semantic priming effects in visual word recognition: A selective
						review of current findings and theories.. Basic progresses in reading. Visual word recognition.

[R38] Neumann O., Prinz W., Sanders A. F. (1984). Automatic processing: A review of recent findings and a plea for
						an old theory.. Cognition and motor processes.

[R39] Neumann O., Klotz W., Umiltá C., Moscovitch M. (1994). Motor responses to nonreportable, masked stimuli: Where is the
						limit of direct parameter specification?. Attention and performance 15: Conscious and nonconscious information
						processing.

[R40] Oldfield R. (1971). The assessment and analysis of handedness: The Edinburgh
						Inventory.. Neuropsychologia.

[R41] Pashler H., Johnston J. C., Ruthruff E. (2001). Attention and performance.. Annual Review of Psychology.

[R42] Posner M. I., Snyder C. R. R., Solso R. L. (1975). Attention and cognitive control..

[R43] Rogers R. D., Monsell S. (1995). Costs of a predictible switch between simple cognitive
						tasks.. Journal of Experimental Psychology: General.

[R44] Rolke B., Heil M., Streb J., Henninghausen E. (2001). Missed prime words within the attentional blink evoke an N400
						semantic priming effect.. Psychophysiology.

[R45] Ruoff A. (1990). Häufigkeitswörterbuch gesprochener
						Sprache.

[R46] Scharlau I., Ansorge U. (2003). Direct parameter specification of an attention shift: Evidence
						from perceptual latency priming.. Vision Research.

[R47] Schneider W., Shiffrin R. M. (1977). Controlled and automatic human information processing: 1.
						Detection, search, and attention.. Psychological Review.

[R48] Valdes B., Catena A., Mari-Beffa P. (2005). Automatic and controlled semantic processing: A masked prime-task
						effect.. Consciousness and Cognition.

[R49] Verleger R., Jaśkowski P., Aydemir A., van der Lubbe R. H., Groen M. (2004). Qualitative differences between conscious and nonconscious
						processing? On inverse priming induced by masked arrows.. Journal of Experimental Psychology: General.

[R50] Vorberg D., Mattler U., Heinecke A., Schmidt T., Schwarzbach J. (2003). Different time courses for visual perception and action
						priming.. Proceedings of the National Academy of Sciences, USA.

[R51] Wolbers T., Schoell E. D., Verleger R., Kraft S., McNamara A., Jaśkowski P. (2006). Changes in connectivity profiles as a mechanism for strategic
						control over interfering subliminal information.. Cerebral Cortex.

